# Case Report: Various Clinical Manifestations Caused by Varicella-Zoster Virus in a Family

**DOI:** 10.3389/fped.2022.876250

**Published:** 2022-06-06

**Authors:** Qinghong Meng, Bingsong Wang, Xianlai Zhang, Zhen Li, Wenjie Wang, Kaihu Yao

**Affiliations:** ^1^Laboratory of Dermatology, Beijing Pediatric Research Institute, Beijing Children’s Hospital, Capital Medical University, National Center for Children’s Health, Beijing, China; ^2^Department of Pediatrics, Wuhu No. 1 People’s Hospital, Wuhu, China; ^3^Department of Laboratory Medicine, Wuhu No. 1 People’s Hospital, Wuhu, China; ^4^Department of Infectious Diseases, The First Affiliated Hospital of Wannan Medical College, Wuhu, China

**Keywords:** varicella-zoster virus, varicella, herpes zoster, breakthrough varicella, atypical

## Abstract

A family cluster of varicella-zoster virus (VZV) infections was reported. Four family members (two children and their parents) continuously develop varicella after the grandmother’s herpes zoster. The unvaccinated 16-month-old infant and his mother developed primary varicella with atypical clinical presentation; however, his 28-year-old father presented with a typical generalized vesicular rash. His vaccinated 4-year-old sister was clinically mild, which could be defined as a breakthrough varicella case. They infected the same virus strain but presented various clinical forms.

## Introduction

Varicella-zoster virus (VZV) infection causes two distinct clinical diseases: varicella (chickenpox) and herpes zoster (shingles) (1). Primary VZV infection causes varicella, which usually occurs in children and presents with a typical generalized vesicular rash (2). Herpes zoster is caused by reactivation of latent VZV in dorsal root ganglia. Herpes zoster occurs most frequently in elders and presents with unilateral dermatomal vesicular rashes (3). VZV is spread by airborne, droplet, and contact transmission.

With the introduction of a 2-dose varicella vaccination program, the incidence of varicella dramatically declined in many industrialized countries (4). The epidemiology of VZV infection was also shifted. In the pre-vaccination era, varicella typically occurs in children, and it plays the most important role in person-person transmission. Herpes zoster appears to be less contagious than varicella (5). In the vaccination era, the incidence of herpes zoster was paradoxically increased (6). Persons with herpes zoster will play a more prominent role in VZV transmission (7). To prevent herpes zoster and reduce the disease burden in the adult population, zoster vaccine has been introduced into a number of countries (8).

In China, a varicella attenuated live vaccine (VarV) immunization of children was started from 1998, with single dose of varicella vaccination administrated at 12 months old. Since 2012, a second dose VarV has been recommended in developed cities and regions, such as Beijing, Shanghai, Zhejiang, Heilongjiang, Shandong, and Fujian provinces (9). However, people received the vaccine at their own expense, and it was not included in the national immunization. Through the National Immunization Program (NIP) monitoring information system of China, the estimated coverage rate of VarV reached to 66.32% in 2014 (10). Zoster vaccine for adults aged ≥50 years was introduced in China to prevent herpes zoster in 2019 (11); however, the coverage was relatively lower. Here, we report a family cluster of VZV infection, which started from a member with herpes zoster, and then presented various clinical forms of varicella in other four members.

## Case Description

**FIGURE 1:**
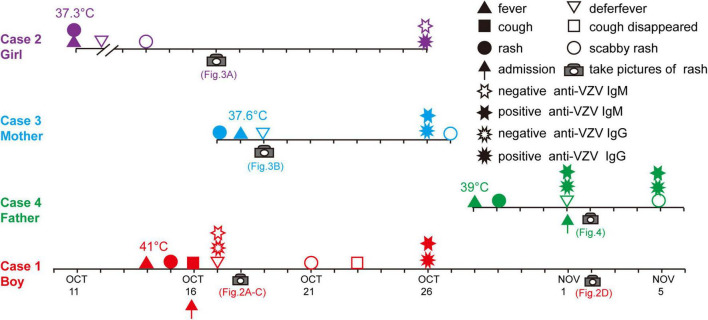
Timeline and key events in the varicella familial cluster.

**FIGURE 2:**
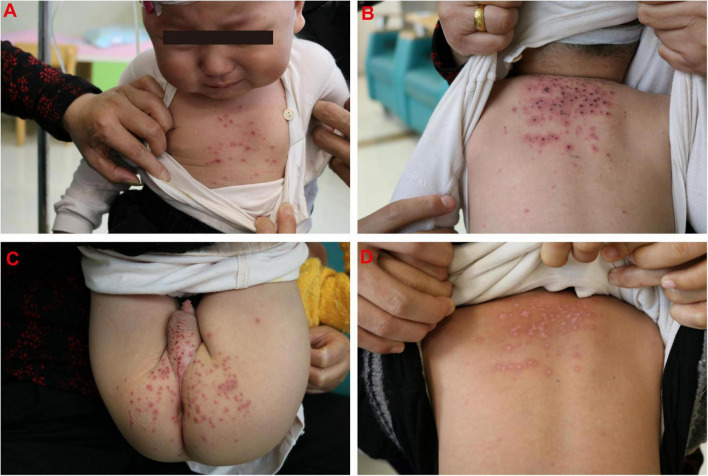
Rash on chest **(A)**, back **(B)**, and hip **(C)** in the boy (case 1). **(D)** A photograph of back showing the rash had disappeared.

The VZV infection order in this family is shown in Figure 1. A 16-month-old immunocompetent boy (the first patient, Case 1) was brought to the hospital with 2 days of fever and 1 day of rash on October 16, 2020. At the current visit, the boy had respiratory symptoms (cough and wet rales), and his temperature was 41°C. He had rash on his chest (Figure 2A), back (Figure 2B), hip (Figure 2C), and sever blisters on the soles of his feet. He was initially diagnosed and treated as hand-foot-and-mouth disease (HFMD), with semi-annual HFMD outbreak in Wuhu, October. However, RT-PCR assay for enterovirus universal primer, Enterovirus 71 (EV71), and Coxsackie A16 (CoxA16) specific primers were negative. Chest radiography performed at the time of admission to our hospital showed multiple nodules coalescing to form nodular consolidation and infiltrates in both lungs. The tests for anti-VZV IgM and IgG were both negative to the October 17 serum sample (Table 1). Treatment with oral acyclovir at a dose of 100 mg two times a day, the boy’s cough resolved on October 23. His serum was taken at the 7-day follow-up visit (October 26), and tested positive for both anti-VZV IgM and IgG (Table 1). The diagnosis of varicella pneumonia was confirmed. His rash completely subsided on November 2 (Figure 2D).

**TABLE 1:** Anti-VZV IgM and IgG results for the family members.

Patient	Time	IgM (U/ml)	IgG (mIU/ml)
Case 1 (Boy)	October 17	8.22 (–)	13.10 (–)
	October 26	195.12 (+)	525.63 (+)
Case 2 (The boy’ sister)	October 26	8.55 (–)	>2000 (+)
Case 3 (The boy’ mother)	October 26	39.28 (+)	878.15 (+)
Case 4 (The boy’ father)	November 1	22.69 (+)	182.57 (+)
	November 5	24.58 (+)	192.38 (+)

*IgM: –, negative (<10 U/ml); +, positive (≥10 U/ml). IgG: –, negative (<50 mIU/ml); +, positive (≥50 mIU/ml).*

**FIGURE 3:**
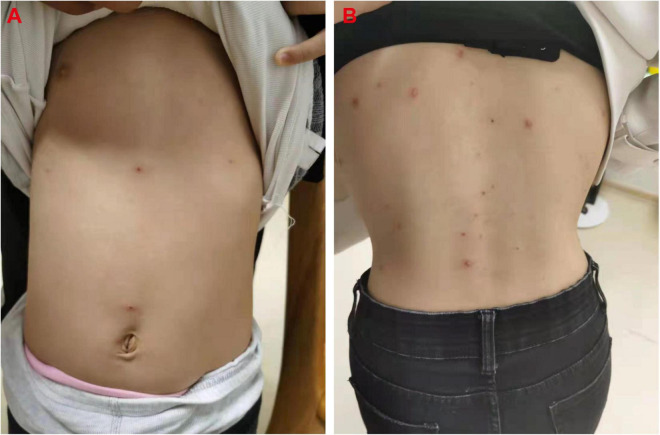
Rash in the girl (case 2, **A**) and the mother (case 3, **B**).

Five days before the boy’s visit (October 11), his 4-year-old immunocompetent sister (the initial varicella patient, Case 2) had developed a low-graded fever and rare rash on her trunk (Figure 3A), and the lesions began crusted on October 14, without seeking any medical care. Her serum was taken at the boy’s 7-day follow-up visit (October 26), and tested negative for anti-VZV IgM but positive for IgG (Table 1). The sister was confirmed that she had received one-dose varicella vaccination 3 years ago at her 14 months of age.

One day after Case 1 admission (October 17), the boy’s 29-year-old mother (Case 3) also developed a rare pruritic rash with vesicles on her trunk (Figure 3B) and a low-graded fever (37.6°C). The mother is a chronic carrier of hepatitis B virus (HBV). Her serum was also taken at the boy’s 7-day follow-up visit (October 26), and also tested positive for both anti-VZV IgM and IgG (Table 1).

**FIGURE 4:**
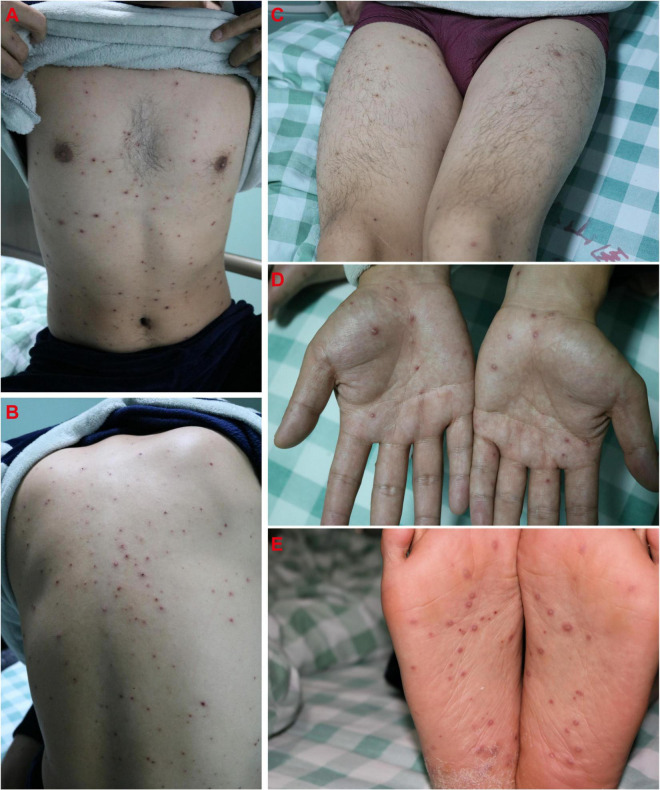
Classic pruritic vesicular rash on trunk **(A–C)** and extremities **(D,E)** in the father (Case 4).

Sixteen days after Case 1 admission (November 1, 2020), the boy’s 28-year-old father (Case 4) was admitted to the hospital because of “4 days of high fever and 3 days of rash.” The father was diagnosed with hepatitis B 3 months ago and received entecavir therapy. Classic pruritic vesicular rash was found on his trunk (Figures 4A–C) and extremities (Figures 4D,E). He was given a diagnosis of varicella on the basis of typical clinical appearance and positive anti-VZV IgM and IgG (Table 1). After acyclovir treatment, his rash began crusted on November 5.

Further investigation revealed the boy’s grandmother had developed herpes zoster 2 weeks (index herpes zoster patient, Case 5) before the illness of the boy’s sister (Case 2). The 57-year-old grandmother suffered from herpes zoster on her chest, back, and axilla. She had no history of tuberculosis, HIV, Hepatitis B or C, or other immunocompromising diseases. She consulted a private Chinese medicine clinic, and her zoster resolved with traditional Chinese medicine treatments for 2 weeks. The grandmother had been taken care of the children and slept with them.

## Discussion

Recently, several VZV family clusters have been reported (12, 13). Different with previous cases that reported the development of varicella in children after exposure to herpes zoster patients, in our case, all the family members (two children and their parents) continuously develop varicella after the grandmother’s herpes zoster infection.

Transmission of varicella from persons with localized herpes zoster most commonly results from direct contact with skin lesions; however, airborne transmission from localized herpes zoster has also been documented in some cases (13–15). The first and most likely scenario of transmission in this family is that the girl was firstly infected by direct contact with skin lesion of the grandmother, as the grandmother took care of these two children and they usually slept together. This scenario shows that vaccinated children could also be infected by localized herpes zoster infection. Breakthrough varicella is generally mild, consistent with the girl. Compared with this boy, even if breakthrough occurs, the benefits of vaccination are evident in this girl. The unvaccinated boy presented with varicella pneumonia. However, before vaccine licensure, varicella is an easily recognized disease, and it is usually diagnosed by the classic clinical presentations. After vaccinations became commonplace, the atypical features of breakthrough varicella, with fewer but more transient lesions that are predominantly maculopapular, make diagnosis more challenging (16).

Breakthrough varicella cases were as contagious as unvaccinated persons with varicella. Therefore, the direct transmission source of the unvaccinated boy is unknown. He could develop the primary varicella from his grandmother or sister. The atypical rash (at HFMD sites) in this boy also led to diagnostic challenges. He received a clinical diagnosis of HFMD at first. On the 2nd day of admission, the mother developed pruritic rash with vesicles, and then the diagnosis of HFMD for the boy was quickly discredited. A clinical diagnosis of varicella was suspected. Laboratory testing is, therefore, increasingly important for diagnosis of VZV infection for confusing or unusual appearing cases (17). The diagnosis of primary varicella for the boy and his mother was confirmed by a positive IgM result or a fourfold increase in IgG titers.

Most adults are not susceptible to varicella (18). Rarely, two adults in this family developed primary varicella, and presented with different clinical forms. They had never been vaccinated against varicella. The clinical presentation of the mother was unusual. Generally, the clinical presentation in adults and immunocompromised subjects is severe and more commonly associated with complications (19), consistent with the father who had chronic hepatitis B.

The incidence of herpes zoster has been increasing worldwide, yet the causation has not been clearly proved. Universal varicella vaccination, aging population and increased prevalence of immunosuppression, and increasing awareness of physicians may contribute to the increased herpes zoster incidence. The total population of China is more than 1.4 billion, and, in 2019, residents aged 50 years and older were estimated to comprise approximately 32% of the population in China. Li et al. estimated that there are 1.563 million new herpes zoster cases annually in mainland China (20). It will play an increasing significant role in VZV transmission. Several cases reported that vaccinated children developed varicella after contacting with their family members with herpes zoster. Widespread use of zoster vaccine could prevent herpes zoster; therefore, it has an impact on reducing the varicella burden. However, when the vaccine price was relatively high, it was not easy to incorporate into a broad NIP, and the coverage was comparatively lower. In addition, recognition of herpes zoster has also important implication for diagnosis of varicella. In the present cases, the recent history of herpes zoster for the grandmother has been acquired after the father’s rash. Consider asking about history of herpes zoster in a family member when a child has atypical or confusing rash.

## Conclusion

The rare family case highlights awareness of various clinical forms of varicella zoster virus infection, importance of laboratory testing for confusing or unusual cases, and recommendation of 2-dose varicella vaccine for children and zoster vaccine for adults.

There are certain limitations in this study. One is a lack of the pictures and clinical situation of the grandmother. Second, no VZV was separated, and DNA information was not confirmed. Third, only a single serum was collected for the sister and the mother.

## Data Availability Statement

The original contributions presented in the study are included in the article/supplementary material, further inquiries can be directed to the corresponding author.

## Ethics Statement

Written informed consent was obtained from the individual(s), and minor(s)’ legal guardian/next of kin, for the publication of any potentially identifiable images or data included in this article.

## Author Contributions

QM and BW conceptualized and designed the study and drafted the initial manuscript. XZ, ZL, and WW designed the data collection instruments and collected data. KY conceptualized and designed the study, coordinated and supervised data collection, and reviewed and revised the original manuscript. All authors contributed to the article and approved the submitted version.

## Conflict of Interest

The authors declare that the research was conducted in the absence of any commercial or financial relationships that could be construed as a potential conflict of interest.

## Publisher’s Note

All claims expressed in this article are solely those of the authors and do not necessarily represent those of their affiliated organizations, or those of the publisher, the editors and the reviewers. Any product that may be evaluated in this article, or claim that may be made by its manufacturer, is not guaranteed or endorsed by the publisher.
